# Safety and Humoral Immunogenicity of Different Dose Levels of Ad26.COV2.S as a 2-Dose Regimen in COVID-19 Vaccine-Naïve Healthy Adults: A Phase 3 Randomized Clinical Trial

**DOI:** 10.3390/vaccines12101136

**Published:** 2024-10-03

**Authors:** Veronica V. Rezelj, Fred Paddenburg, Marie Enajite Diegbe, Julius Nangosyah, Emil C. Reisinger, Weihong Hu, Carla Truyers, Gert Scheper, Mathieu Le Gars, Jenny Hendriks, Frank Struyf, Macaya Douoguih, Hanneke Schuitemaker, Javier Ruiz-Guiñazú

**Affiliations:** 1 Janssen Vaccines & Prevention, 2301 CN Leiden, The Netherlands; veronica.x.rezelj@gsk.com (V.V.R.); gscheper@its.jnj.com (G.S.); mathieu.legars1@gmail.com (M.L.G.); jhendri1@its.jnj.com (J.H.); macaya.douoguih@merck.com (M.D.); hanneke.schuitemaker@valneva.com (H.S.); 2 Vimef Holding B.V., 3311 GE Dordrech, The Netherlands; fred.paddenburg@gmail.com; 3 Cytel Global Headquarters, Cambridge, MA 02139, USA; julius.nangosyah@cytel.com (J.N.); whu38@its.jnj.com (W.H.); 4 Rostock University Medical Center, 18057 Rostock, Germany; emil.reisinger@uni-rostock.de; 5 Janssen Research and Development, 2340 Beerse, Belgium; ctruyers@its.jnj.com (C.T.); fstruyf@gmail.com (F.S.)

**Keywords:** Ad26.COV2.S, vaccine, COVID-19, adult, immunogenicity, spike-binding antibodies, safety, neutralizing antibodies

## Abstract

Background: This study aimed to support the end-of-shelf life specification (2.5 × 10^10^ virus particles [vp]) for the standard Ad26.COV2.S dose (5 × 10^10^ vp). Methods: This randomized, double-blind Phase 3 study evaluated immunogenicity, reactogenicity, and safety of several Ad26.COV2.S dose levels (range 1.25 to 9 × 10^10^ vp) in 1593 adults between June 2021 and July 2023. Results: Spike-binding antibody responses 28 days post-dose 1 were non-inferior for the 9 × 10^10^ vp, but not the 2.5 × 10^10^ vp group when compared with the standard dose. Non-inferiority was demonstrated in terms of spike-binding antibody responses 14 days post-dose 2 for each dose level, including the lowest dose level of 1.25 × 10^10^ vp, compared to 28 days after one dose and 14 days after two doses of the standard dose. Spike-binding antibody levels correlated well with virus neutralizing titers. There was no impact of pre-existing Ad26.COV2.S neutralizing titers on immunogenicity at any dose level. All dose levels were well tolerated. Conclusions: This study highlights the challenges associated with conducting clinical studies in a rapidly evolving environment and underscores the importance of platform data that can guide initial vaccine specifications such as shelf life during accelerated vaccine development. The present study supports the end-of-shelf life specifications for the approved Ad26.COV2.S dose, and could provide useful information in future vaccine developments using adenovirus vector vaccines.

## 1. Introduction

Ad26.COV2.S is a recombinant, replication-incompetent human adenovirus type 26 (Ad26) vector coronavirus disease 2019 (COVID-19) vaccine that encodes the SARS-CoV-2 spike protein in its prefusion conformation. In 2021, Ad26.COV2.S was granted emergency use authorization by the United States Food and Drug Administration and conditional marketing authorization by the European Commission. It was subsequently Authorized or conditionally approved in more than 120 countries/territories worldwide. Ad26.COV2.S is administered as a single priming dose to adults aged 18 years and older [1]. In Phase 3 trials, one dose of Ad26.COV2.S was effective in preventing moderate-to-severe/critical COVID-19, as well as hospitalizations and deaths due to COVID-19 [2,3].

Each dose of Ad26.COV2.S contains 5 × 10^10^ virus particles (vp). However, the concentration of vp may vary slightly across batches at the time of release due to the manufacturing process. Titers also decline over the shelf life of a vaccine, which includes the time taken to pack, label, transport, and store the vaccine until use. Vaccine shelf life refers to an expiry date after which the potency of the vaccine may no longer be sufficient to induce an adequate immune response. Shelf life is determined by stability studies. The minimal release concentration is that which ensures the potency of the vaccine throughout its shelf life.

At the end of 2020 and in early 2021, the pressure to register and deliver COVID-19 vaccines was at its height. Accelerated vaccine development posed significant challenges for estimation of shelf life due to the short interval available for stability studies between Phase 1 studies and regulatory submission [4]. In such settings, data from other approved vaccines manufactured using the same platform, can provide invaluable information for guiding estimations of shelf life and optimal release titer. Initial specifications for Ad26.COV2.S were modeled on previous experience with the Ad26.ZEBOV/Zabdeno Ebola vaccine with incorporation of COVID-19-specific data as they became available [4]. The first lot specifications for Ad26.COV2.S were initially based on studies in non-human primates. At that time, the efficacy limit (or end of shelf life titer) was 2.5 × 10^10^ vp, and the estimated initial shelf life was around 4.5 months, subsequently confirmed using Phase 2 immunogenicity and Phase 3 efficacy data [4]. The shelf life was extended to 24 months at −25 to −15 °C, of which 11 months could be at 2 to 8 °C, as additional data became available [1].

Clinical evaluation of shelf life aims to assess immunogenicity and safety of reducing titers to identify the lower limit of clinical potency. Likewise, evaluation of the safety of higher titers that reflect the maximum expected when a batch is released, or to support an increase in the shelf life, is also important [5].

We assessed the immunogenicity, reactogenicity, and safety of a range of Ad26.COV2.S dose levels by assessing non-inferiority (NI) of immune responses relative to the approved dose level (5 × 10^10^ vp) for use in adults. The purpose of the study was to aid in the establishment of end-of-shelf life specifications for Ad26.COV2.S after conditional approval of the vaccine and its initial release protocols. Lower dose levels (3.5 × 10^10^ vp, 2.5 × 10^10^ vp, and 1.25 × 10^10^ vp) were tested to mimic vaccine degradation to enable determination of product expiry. A potential target release titer of 7 × 10^10^ vp was evaluated in order to potentially lengthen shelf life, and a higher titer of 9 × 10^10^ vp was also evaluated as the upper limit of the release range (potential maximum number of vp at release).

## 2. Materials and Methods

### 2.1. Study Design, Vaccine, and Participants

This randomized, double-blind Phase 3 study was conducted in the United States (15 centers), Brazil (10 centers), Germany (5 centers), and South Africa (3 centers) (NCT04908722). Eligible participants were healthy adults aged between 18 and 55 years who had no history of COVID-19 vaccination. Participants were excluded if they had abnormal immune system functioning, a history of capillary leak syndrome, a history of thrombosis and thrombocytopenia syndrome (TTS), heparin-induced thrombocytopenia, or thrombosis. Individuals with medical conditions such as chronic diseases that increased the risk of progression to severe COVID-19 were excluded. A full list of inclusion and exclusion criteria is provided in the Supplementary Materials.

**Figure 1:**
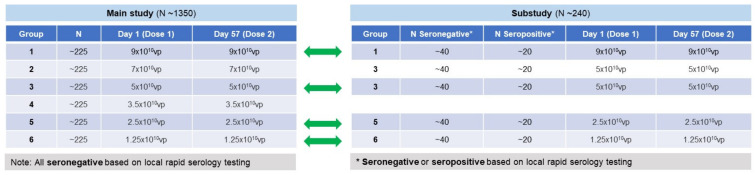
Study groups and design overview.

There were 2 parts to the study: participants in the main study (Part 1) were randomized (1:1:1:1:1:1) to receive 1 of 6 dose levels of Ad26.COV2.S (9 × 10^10^ vp, 7 × 10^10^ vp, 5 × 10^10^ vp [clinical dose], 3.5 × 10^10^ vp, 2.5 × 10^10^ vp, and 1.25 × 10^10^ vp) administered as a 2-dose schedule (56-day interval) (Figure 1). The co-primary objectives of Part 1 were to demonstrate NI of spike-binding antibody responses after 1 and 2 doses of each dose level versus 1 or 2 doses of 5 × 10^10^ vp (clinical dose) in SARS-CoV-2-seronegative adults. Assessment of vaccine immunogenicity endpoints and safety were secondary objectives.

Part 2 was a sub-study to further characterize the innate, pro-inflammatory, and other relevant (e.g., pro-thrombotic) responses to Ad26.COV2.S to better understand the potential association with TTS [6]. Eligibility criteria were the same as the main study except that a positive diagnostic test for past SARS-CoV-2 testing did not exclude participation in the sub study up to a target of 20 seropositive participants per group. Participants enrolled in the sub-study were randomized (1:1:1:1) to receive 2 doses of Ad26.COV2.S (56-day interval) at doses of 9 × 10^10^ vp, 5 × 10^10^ vp (clinical dose), 2.5 × 10^10^ vp, or 1.25 × 10^10^ vp (Figure 1). The results of Part 2 will be published separately.

All participants were screened for previous SARS-CoV-2 infection by locally performed rapid finger prick serology. All participants in the main study were to be SARS-CoV-2 seronegative at enrollment, while in the sub-study, approximately two-thirds of participants were to be seronegative and one-third seropositive, as determined by finger-prick serology.

All vaccines were given intramuscularly into the deltoid. Participants were followed up for immunogenicity for 6 months. Safety was initially assessed until 12 months after the last active vaccination but was reduced to 6 months following a protocol amendment.

The study was reviewed and approved by national, regional, or institutional review boards or independent ethics committees. All participants provided written informed consent before enrollment.

### 2.2. Randomization and Blinding

Randomization used an Interactive Web Response system and was balanced by using randomly permuted blocks. Participants and investigators were blinded to the study group throughout the study. Blinding was guaranteed by the preparation of vaccine by an unblinded study vaccine administrator using masked syringes. The sponsor and statisticians were unblinded at primary analysis when all participants completed the Day 85 visit or had discontinued the study. Global unblinding occurred when all participants had either discontinued or completed the last visit.

### 2.3. Study Endpoints

**Figure 2:**
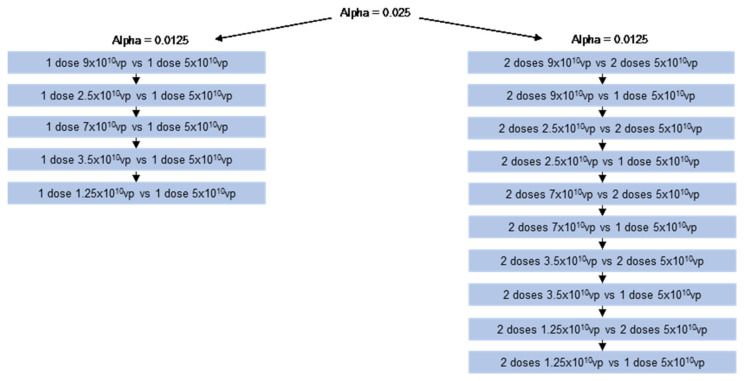
Tree-based schema for testing post-dose 1 and post-dose 2 NI hypotheses. Each post-dose 1 (**left**) and post-dose 2 (**right**) hypothesis is represented by a blue box. Hypothesis testing followed a sequential approach, starting with the highest dose tested first. The family-wise error rate was controlled at α = 0.025 (1-sided). The 2-sided (1 − α)% confidence interval for the difference in means was constructed based on the sampling distribution of 2 independent normal distributions with variances that were unknown but assumed to be equal.

The primary endpoint was the binding antibody responses to SARS-CoV-2 S protein for each dose level at 28 days after the first vaccination or 14 days after the second vaccination (Figure 2). NI was demonstrated by the ratio of the geometric mean concentrations (GMCs) for each study dose level relative to the 5 × 10^10^ vp dose: if the 97.5% confidence interval (CI) of the GMC ratio at each time point was entirely above the pre-specified NI margin of 0.67.

Endpoints to support secondary immunogenicity objectives were binding antibody responses at other time points and neutralizing antibody levels. Correlations between spike-binding antibodies and neutralizing antibodies were explored, as well as investigation of the impact of pre-existing Ad26 antibodies on vaccine immunogenicity.

Solicited adverse events (AEs) were recorded for 7 days after each vaccination, unsolicited AEs for 28 days after each vaccination, medically attended AEs (MAAEs) until 6 months post dose 2, and serious AEs (SAEs) and adverse events of special interest (AESIs) until study end. AE severity was graded according to the United States Food and Drug Administration guidance [7].

### 2.4. Immunogenicity Evaluation

SARS-CoV-2 spike binding-antibody levels were measured prior to each vaccination (Days 1 and 57), 28 days after dose 1 (Day 29), 14 days after dose 2 (Day 71), and at Weeks 32 and 60 (i.e., approximately 6 and 12 months post-last vaccination, respectively) using a validated human SARS-CoV-2 pre-spike-specific IgG enzyme-linked immunosorbent assay (S-ELISA) [8]. SARS-CoV-2 nucleocapsid protein (N)-specific binding antibodies were measured using the validated Roche Elecsys^®^ assay (by Labcorp) at Days 1 (baseline), 29, 71, and at Weeks 32 and 60, to identify participants with SARS-CoV-2 infections (defined as N-serology seroconversion) during the study.

Neutralizing antibodies against the reference strain were measured by a pseudotyped virus neutralization assay (psVNA) performed by Monogram Laboratories in a subset of participants from the main study (100 participants per group) and all participants in the sub-study. The assay is based on pseudovirions that express the SARS-CoV-2 S protein from the Wuhan strain (with a D614G mutation).

Neutralizing antibodies against the Ad26 vector backbone were measured in all participants in the sub-study, and 25 randomly selected samples from each group of the main study on Day 1 and Day 57 using an adenovirus neutralization assay at Nexelis laboratories (Ad26 VNA), as previously described [9]. Detailed assay methods are provided in the Supplementary Information.

Serostatus at baseline was determined based on central, validated assays (S-ELISA and N-serology), with a participant declared seropositive if at least 1 of those results was positive (>lower limit of quantification (LLOQ)), and seronegative if both results were negative.

### 2.5. Safety and Reactogenicity Evaluation

Solicited AEs were recorded on electronic diary cards and were evaluated by the investigator. MAAEs were defined as AEs requiring visits to a hospital, emergency room, urgent care clinic, or other visits to or from medical personnel for any reason and included the new onset of chronic diseases. The AESI was TTS and all thrombotic or thrombocytopenia events were considered suspected AESI [10]. Any case of co-occurring thrombosis and thrombocytopenia was referred to an AESI Adjudication Committee with appropriate expertise to determine whether it was a case of TTS.

Other safety assessments included clinical laboratory assessments including platelet count measurements, vital signs measurements (pulse/heart rate, supine systolic and diastolic blood pressure, respiratory rate, and body temperature) and periodic physical examination.

### 2.6. Statistical Analysis

The originally intended population for NI testing was participants who were SARS-CoV-2 seronegative at baseline and who did not become infected with SARS-CoV-2 during the study (NI analysis set). However, an unexpectedly high number of N-serology positive results were observed in the main study reflecting either high infectious pressure and/or insufficient sensitivity of the local finger prick serology tests, reducing the power of the study to conclude NI. Consequently, NI was evaluated on the per protocol immunogenicity (PPI) analysis set on the combined populations from the main study and sub-study. The analysis model accounted for SARS-CoV-2 serostatus at baseline as a covariate.

The PPI population included all randomized and vaccinated participants for whom immunogenicity data were available, excluding participant samples after protocol deviations expected to impact immunogenicity outcomes and those after SARS-CoV-2 infections. The NI population included was a subset of the PPI and included only participants who were SARS-CoV-2 negative at baseline.

NI hypotheses for post-dose 1 and post-dose 2 were tested separately to control the family-wise error rate at alpha = 0.025 (1-sided) (Figure 2). Post-dose 1 NI comparisons assessed spike-binding antibody GMCs 28 days after dose 1. Post-dose 2 assessments compared GMCs 14 days after the second vaccination at each dose group level versus the 5 × 10^10^ vp group 28 days after the first vaccination, and versus the 5 × 10^10^ vp group 14 days after the second vaccination.

Post-dose 1 and post-dose 2 NI hypothesis testing followed a sequential approach, starting with the highest dose tested first (Figure 2). The 2.5 × 10^10^ vp dose level was tested ahead of the 7 × 10^10^ vp and 3.5 × 10^10^ vp groups as the latter two arms were not included in the sub-study and consequently had smaller sample sizes.

Descriptive statistics (geometric means, geometric means of fold increase, and corresponding 95% CIs) were performed for the PPI and NI populations. For N-serology-negative participants with missing N-serology results prior to their first positive N-serology result, data from the time points with missing N-serology status were excluded from the analysis to exclude potential SARS-CoV-2 infections.

Results from the Part 2 sub-study relating to the pro-inflammatory response will be published separately. The analysis was performed using SAS Studio 3.8.

## 3. Results

### 3.1. Study Participants and SARS-CoV-2 Serostatus at Baseline

**Figure 3:**
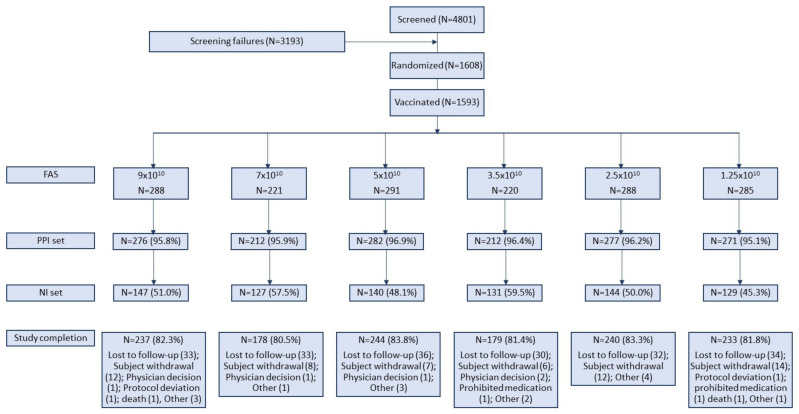
Participant flow (main and sub-studies combined). Abbreviations: FAS, full analysis set; PPI, per protocol immunogenicity; N, number of participants; NI, non-inferiority.

The study was conducted between June 2021 and July 2023. A total of 1593 participants were randomized and received at least one dose of Ad26.COV2.S (Figure 3). Participants were enrolled in Brazil (50.9%), Germany (23.1%), the US (18.3%) and South Africa (7.7%). The average follow-up duration was 298.3 days. The mean age (standard deviation) of participants was 34.7 (9.81) years; 62.9% were male, 60.3% were White, and 31.5% were Black or African American. There were 282 (17.7%) participants who discontinued the study prematurely, mainly due to loss to follow-up or withdrawal by the participant. Thirteen participants discontinued vaccination due to AEs, all had COVID-19, and one additionally reported vaccination site erythema. Three COVID-19 cases and the case of erythema were considered by the Investigator to be related to vaccination.

Preliminary blinded evaluation of central baseline N-serology results identified a high number of initially seropositive participants in the main study. At the final analysis, 46.0% of participants in the main and sub-studies were SARS-CoV-2 seropositive at baseline.

Baseline characteristics were generally well-balanced across the vaccination groups (Table 1). Because the 7 × 10^10^ vp and 3.5 × 10^10^ vp groups were not included in the sub-study, where a proportion of seropositive participants was enrolled, the percentage of seropositive participants in these two groups was lower (approximately 38%) compared to between 46 and 52% in other groups.

### 3.2. Non-Inferiority

**Figure 4:**
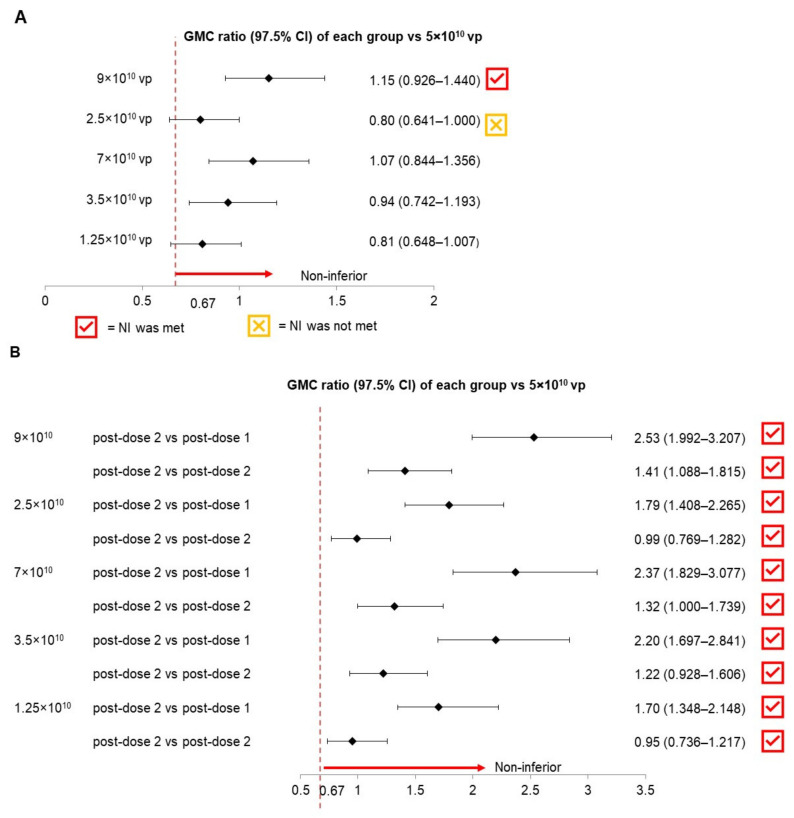
Comparison of SARS-CoV-2 spike-binding antibody levels following vaccination with different dose levels of Ad26.COV2.S versus 5 × 10^10^ vp (**A**) GMC ratios of each dose level versus 5 × 10^10^ vp 28 days post-dose 1 (**B**) GMC ratios of each dose level 14 days post-dose 2 versus 5 × 10^10^ vp dose level either 28 days post-dose 1 or 14 days post-dose 2 (per protocol immunogenicity analysis set). Abbreviations: CI, confidence interval; GMC, geometric mean antibody concentration; vp, viral particles. The dotted red line represents the NI margin of 0.67 for the lower limit of the GMR 97.5% CI (2-sided).

At 28 days post-dose 1, NI was demonstrated for the 9 × 10^10^ vp group compared with the 5 × 10^10^ vp group, but not the 2.5 × 10^10^ vp group compared with the 5 × 10^10^ vp group (Figure 4A). The 7 × 10^10^ vp and 3.5 × 10^10^ vp dose levels met the success criterion although NI could not be concluded because of the sequential testing strategy.

NI was demonstrated in terms of spike-binding antibody responses 14 days post-dose 2 for each dose level, including the lowest dose level of 1.25 × 10^10^ vp, compared to 28 days post-1 dose of 5 × 10^10^ vp Ad26.COV2.S, and versus 14 days post-2 doses of 5 × 10^10^ vp Ad26.COV2.S (Figure 4B).

### 3.3. Humoral Immunogenicity

**Figure 5:**
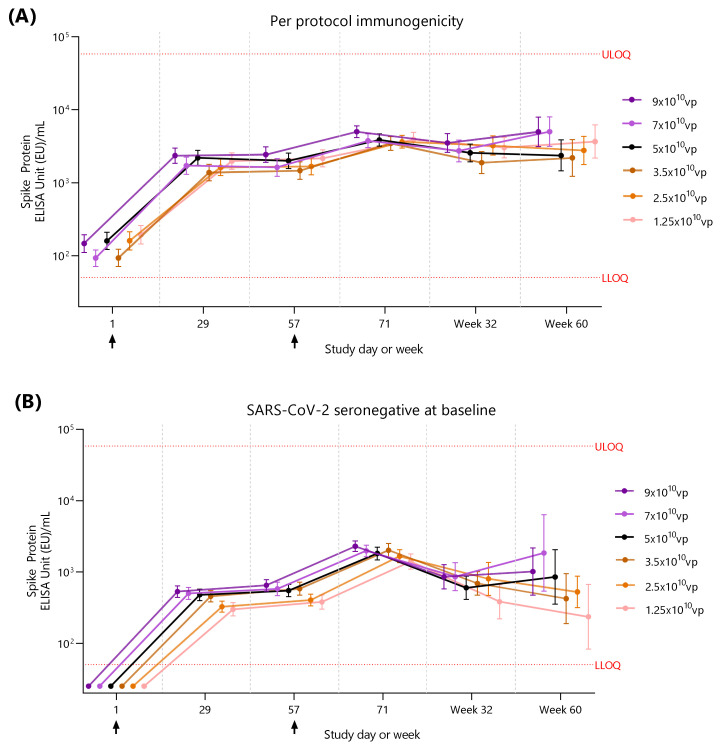
SARS-CoV-2 spike-binding antibody levels following 2 doses of Ad26.COV2.S at different dose levels (**A**) per-protocol immunogenicity analysis set, and (**B**) non-inferiority analysis set. Geometric mean concentrations (GMCs) and 95% confidence intervals are shown as dots and error bars. The *x*-axis is not continuous. Black arrows indicate Ad26.COV2.S vaccination, and red lines indicate the assay LLOQ and ULOQ (50.3 EU/mL and 58,158.1 EU/mL, respectively). Data are tabulated in Tables S1 and S2.

All Ad26.COV2.S dose levels induced spike-binding antibody responses after the first dose, with a further increase in titers after dose 2. In the PPI population, spike-binding antibody levels increased between 7.3- and 12.7-fold from baseline at Day 29 (Table S1). The Day 29 GMCs (EU/mL) were 2351 in the 9 × 10^10^ vp group, 1714 in the 7 × 10^10^ vp group, 2189 in the 5 × 10^10^ vp group, 1377 in the 3.5 × 10^10^ vp group, 1626 in the 2.5 × 10^10^ vp group, and 1976 the 1.25 × 10^10^ vp group (Figure 5A).

Spike-binding antibodies were sustained through 56 days post-vaccination and further increased between 13- and 30.3-fold from baseline by Day 71 (14 days after dose 2) (Table S1). Spike-binding antibody levels remained well above baseline levels 6 and 12 months after dose 2.

Similar results were observed for neutralizing antibodies against the SARS-CoV-2 reference strain in a subset of participants (Table S3, Figure S1A).

A high correlation between binding and neutralizing antibodies was found at all time points (Spearman’s correlation coefficient > 0.82 at all time points) (Figure S2).

#### Immunogenicity in Participants Seronegative for SARS-CoV-2 at Baseline

Spike-binding antibody GMCs were lower in baseline-seronegative participants than in the PPI population, and a dose-dependent response was observed, with higher binding antibody levels elicited by higher dose levels (Figure 5B). Day 29 GMCs (EU/mL) post-dose 1 were 530 in the 9 × 10^10^ vp group, 502 in the 7 × 10^10^ vp group, 479 in the 5 × 10^10^ vp group, 456 in the 3.5 × 10^10^ vp group, 326 in the 2.5 × 10^10^ vp group, and 300 in the 1.25 × 10^10^ vp group (Table S2).

Spike-binding antibody levels remained well above baseline until 12 months post-dose 2. Increased variability in the responses from 6 months post-dose 2 reflects the decreasing number of participants in this cohort over the study period (Table S2).

Neutralizing antibody responses followed similar trends to those described for binding antibodies (Figure S1B, Table S4).

### 3.4. Impact of Pre-Existing Anti-Vector Antibodies on Spike-Binding Antibody Responses

**Figure 6:**
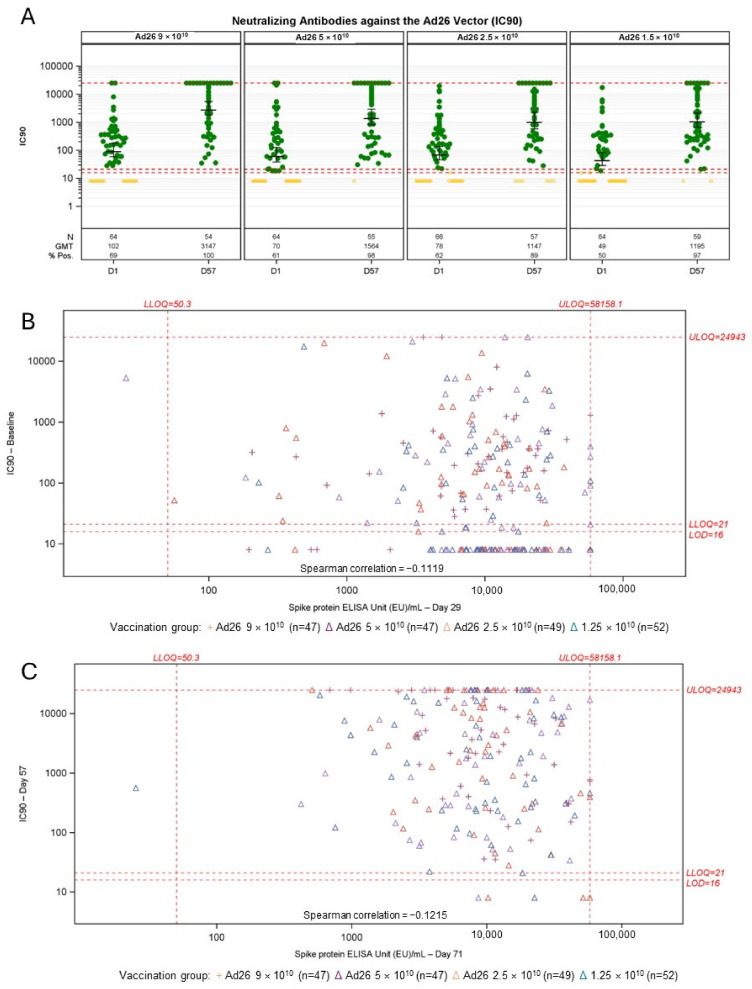
Impact of pre-existing adenovirus 26 (Ad26) neutralizing antibody titers on spike-binding antibody responses elicited by Ad26.COV2.S. (**A**) Anti-Ad26 neutralizing antibody titers prior to each vaccination (Day 1 and Day 57). Greed dots are positive samples, yellow dots are negative samples. 95% confidence intervals are calculated on positive samples, (**B**) correlation between pre-existing anti-Ad26 neutralizing antibody and spike-binding antibodies post-dose 1 (Day 29), (**C**) correlation between anti-Ad26 neutralizing antibody titers prior to dose 2 (Day 57) and spike-binding antibody titers 14 days post-dose 2 (Day 71). IC90—90% inhibitory concentration; LLOQ, lower limit of quantification; N, number of subjects with data; ULOQ, upper limit of quantification; vp, viral particles. Neutralizing titers were measured on all participants in the sub-study and 25 from each group in the main study.

Neutralizing antibodies against Ad26 were assessed in participants in the sub-study. Between 50% and 69% of pre-vaccination serum samples in each dose group had detectable neutralizing antibodies against Ad26, with geometric mean titers (GMTs) between 49 and 102 IC90 (Figure 6A). On Day 57, the percentage with detectable Ad26 neutralizing antibodies had increased to 89% to 100%. GMTs increased to between 1147 and 3147 IC90, with a trend for higher dose levels to induce higher responses. Across all dose levels, the correlation of Ad26 VNA titers prior to vaccination 1 and spike-specific binding antibody titers 28 days post vaccination 1 was low (Spearman correlation coefficient = −0.1119) (Figure 6B). Similarly, there was poor correlation between pre-dose 2 Ad26 VNA titers and spike-binding antibodies 14 days post-dose 2 (Spearman correlation coefficient = −0.1215) (Figure 6C).

### 3.5. Reactogenicity and Safety

All vaccine doses were well tolerated. Vaccination site pain was the most frequently reported solicited local AE in all groups after each dose (Table 2). Pain was reported by between 46.0% (1.25 × 10^10^ vp group) and 64.3% (7 × 10^10^ vp group) of participants after dose 1, and between 39.9% (1.25 × 10^10^ vp group) and 50.0% (7 × 10^10^ vp group) after dose 2. Incidences of pain were lowest in the lowest dose group; however, there was no consistent dose effect on the incidence of severity of local AEs after either dose. Grade 3 local AEs were uncommon, reported by no more than 2.3% of participants in any group after both doses.

Fatigue, headache, and myalgia were the most frequently reported solicited systemic AEs in each group (Table 3). Fatigue was reported by between 35.4% (2.5 × 10^10^ vp group) and 57.5% (7 × 10^10^ vp group) of participants across dose levels, headache by between 35.4% (1.25^10^ vp group) and 57.5% (7 × 10^10^ vp group), and myalgia by 31.9% (1.25 × 10^10^ vp group) and 54.5% (9 × 10^10^ vp group) after dose 1. After dose 2, between 26.3% (2.5 × 10^10^ vp group) and 37.5% (7 × 10^10^ vp group) of participants reported fatigue, 24.8% (1.25 × 10^10^ and 2.5 × 10^10^ vp groups) to 37.9% (9 × 10^10^ vp group) reported headache, and 23.3% (1.25 × 10^10^ vp group) to 39.0% (9 × 10^10^ vp group) reported myalgia. Systemic AEs (any and grade 3) tended to occur more frequently in the higher dose ranges (7 × 10^10^ vp and 9 × 10^10^ vp groups).

The most frequently reported Grade 3 systemic AEs were fatigue, reported by between 3.1% (2.5 × 10^10^ vp group) to 9.5% (7 × 10^10^ vp group) of participants, headache, reported by between 0% (5 × 10^10^ vp group) and 7.2% % (7 × 10^10^ vp group), and myalgia, reported by between 1.4% (2.5 × 10^10^ vp group and 1.25 × 10^10^ vp groups) and 6.3% (7 × 10^10^ vp), after dose 1. After dose 2, between 0.7% (2.5 × 10^10^ vp group) and 2.5% (7 × 10^10^ vp group) of participants reported Grade 3 fatigue, 0% (1.25 × 10^10^) to 3.8% (9 × 10^10^ vp group) reported Grade 3 headache, and 0.4% (5 × 10^10^ vp group) to 1.5% (2.5 × 10^10^ vp group) reported myalgia.

The median duration of each solicited local and systemic AE was 1–3 days. The incidence and severity of local and systemic AEs decreased after the second dose.

The percentage of participants in each group who reported at least 1 unsolicited AE ranged from 18.9% (5 × 10^10^ vp group) to 27.1% (9 × 10^10^ and 7 × 10^10^ vp groups) after dose 1, and 14.0% (7 × 10^10^ vp group) to 20.8% (9 × 10^10^ vp group) after dose 2. Considering both doses combined, the most frequently reported unsolicited AEs were influenza and headache, each reported by 6.3% of participants (Table S5).

Two participants died during the study: one drowned 99 days after dose 1 (9 × 10^10^ vp group), and an unknown cause of death was reported for the other 313 days after dose 2 (1.25 × 10^10^ vp group). Neither death was considered to be related to the study vaccination by the Investigator.

There were 32 participants who reported SAEs during the study (Table S6), none of which was reported for more than 1 participant, except for spontaneous abortion (N = 2). None of the SAEs were considered by the Investigator to be related to vaccination.

Suspected AESIs (either thrombosis and/or thrombocytopenia) were reported for 33 participants overall. There were no cases of co-occurring thrombosis and thrombocytopenia and therefore no cases qualified for TTS assessment by the adjudication committee.

MAAEs were reported in 256 participants, most frequently for influenza and COVID-19 (42 participants each). All other MAAEs were reported in fewer than 1% of participants. The frequency and type of SAEs, AESIs, and MAAEs had a similar distribution across dose levels over the study period.

## 4. Discussion

This study was conducted during the SARS-CoV-2 pandemic during a period of rapid change in levels of population immunity to SARS-CoV-2. The assumptions underpinning the primary NI analysis was based on SARS-CoV-2 seronegative participants. Low sensitivity of the screening finger prick test, since reported by others [11,12], combined with the rapidly evolving pandemic, meant that evaluation of the primary objective in seronegative participants became unfeasible. In response, before data unblinding, the pre-specified analyses were modified to be conducted on seropositive and seronegative participants, fitting the NI model with dose level as the independent variable, and adjusting for SARS-CoV-2 serostatus at baseline.

The specified end-of-shelf life titer of 2.5 × 10^10^ vp marginally failed to meet the NI criterion post-dose 1. While we adjusted for baseline serostatus, we could not account for other variables not included in the randomization algorithm, such as the timing and number of previous SARS-CoV-2 infections, and infecting variant(s) which could introduce variability in terms of the humoral immune response to vaccination in the tested population. Such variability was not accounted for in the sample size assumptions and likely explains the marginal failure of the 2.5 × 10^10^ vp dose level, whereas the 1.25 × 10^10^ vp dose level, which is equivalent to half the dose of 2.5 × 10^10^ vp, showed a similar GMR to the 2.5 × 10^10^ vp dose level (0.81 vs. 0.80, respectively) but did not pass the NI assessment.

Consistent with this hypothesis, dose-dependent binding and neutralizing antibody levels were observed in participants who were seronegative at baseline, whereas this trend was not consistent in the PPI analysis set of whom 46.0% were SARS-CoV-2 seropositive at baseline. The data do not indicate any clinically significant difference in spike-binding antibody responses between the 2.5 × 10^10^ vp and 5 × 10^10^ vp dose groups in baseline seronegative participants, supporting the end-of-shelf life specification.

There was no impact of pre-existing neutralizing antibodies against Ad26 on vaccine-induced immune responses at any dose level, which is consistent with studies of other vaccines using the Ad26 vector platform (Ebola, human immunodeficiency virus, Zika, and COVID-19) [8,13,14,15,16].

All Ad26.COV2.S dose levels were well tolerated. The incidence of solicited local and systemic AEs appeared lower at lower dose levels, but no consistent dose-related trend was observed in this population. A previous dose-finding study in SARS-CoV-2 seronegative adults reported dose-dependent reactogenicity after Ad26.COV2.S [17]. The heterogenous baseline serostatus of the FAS population used for the safety analysis may have obscured similar findings in our study [17]. Incidences and severity of AEs were aligned with the established reactogenicity profile of the approved 5 × 10^10^ vp dose [1]. Reactogenicity decreased after the second dose. Potential AESIs, SAEs, and vaccine discontinuations were similar across dose groups. No new safety signals were identified in this study.

Few dose ranges of adenovirus vector vaccines have been conducted. A recombinant adenovirus type-5 (Ad5)-vectored COVID-19 vaccine expressing the Spike glycoprotein (5 × 10 ^10^, 1 × 10 ^11^ and 1.5 × 10 ^11^ vp) induced dose-dependent immunogenicity and reactogenicity in initially seronegative participants [18]. In a Phase 2 study of the same vaccine, the 5 × 10 ^10^ vp vaccine formulation induced generally comparable immunogenicity and an improved tolerability profile versus a 1 × 10 ^11^ dose, leading to the selection of the 5 × 10 ^10^ vp dose for further development [19]. Both studies showed an adverse impact of pre-existing Ad5 neutralizing antibodies on immune responses.

This dose-finding study highlights the challenges of conducting clinical trials during a rapidly evolving public health emergency. Platform data from other Ad26.COV2.S-based vaccine developments were critical in supporting early vaccine release specifications and were later confirmed by clinical data using Ad26.COV2.S. Platform development is a useful strategy during accelerated vaccine development [4]. Data from the current study contribute to the body of data around Ad26-vector vaccines and may be useful in future emergency settings to help guide and support accelerated vaccine development.

## 5. Conclusions

In conclusion, this study supports the end-of shelf life titer for Ad26.COV2.S initially determined from platform and non-clinical data. Vaccination induced dose-dependent binding and neutralizing antibody levels in initially seronegative, but not initially positive, participants. The presence of anti-vector antibodies did not impact the immune response to vaccination. While the incidences of some solicited AEs were observed to be higher in the higher dose levels, the reactogenicity profile was aligned with the approved 5 × 10^10^ vp dose. These data could help inform future accelerated vaccine developments using adenovirus vectors.

## Figures and Tables

**Table 1:** Summary of demographics and baseline characteristics (Full analysis set—main and sub study).

	Ad26.COV2.S 9 × 10^10^ vp	Ad26.COV2.S 7 × 10^10^ vp	Ad26.COV2.S 5 × 10^10^ vp	Ad26.COV2.S 3.5 × 10^10^ vp	Ad26.COV2.S 2.5 × 10^10^ vp	Ad26.COV2.S 1.25 × 10^10^ vp
N	288	221	291	220	288	285
Age, years, mean (SD)	35.6 (10.21)	35.2 (9.87)	34.8 (9.82)	34.7 (9.36)	34.5 (9.81)	33.4 (9.63)
18–40 years	189 (65.6%)	159 (71.9%)	205 (70.4%)	161 (73.2%)	213 (74.0%)	219 (76.8%)
41–55 years	99 (34.4%)	62 (28.1%)	86 (29.6%)	59 (26.8%)	75 (26.0%)	66 (23.2%)
Sex						
Male	170 (59.0%)	148 (67.0%)	172 (59.1%)	148 (67.3%)	182 (63.2%)	182 (63.9%)
Female	117 (40.6%)	73 (33.0%)	119 (40.9%)	72 (32.7%)	106 (36.8%)	103 (36.1%)
Undifferentiated	1 (0.3%)	0	0	0	0	0
Race						
American Indian/Alaska Native	1 (0.3%)	1 (0.5%)	3 (1.0%)	1 (0.5%)	0	0
Asian	3 (1.0%)	3 (1.4%)	4 (1.4%)	3 (1.4%)	2 (0.7%)	4 (1.4%)
Black or African American	104 (36.1%)	57 (25.8%)	90 (30.9%)	63 (28.6%)	87 (30.2%)	101 (35.4%)
Native Hawaiian/other Pacific Islander	1 (0.3%)	0	1 (0.3%)	0	1 (0.3%)	0
White	161 (55.9%)	142 (64.3%)	174 (59.8%)	138 (62.7%)	183 (63.5%)	163 (57.2%)
Unknown/not reported	16 (5.5%)	17 (7.7%)	15 (5.2%)	11 (5.0%)	12 (4.2%)	15 (5.3%)
Multiple	2 (0.7%)	1 (0.5%)	4 (1.4%)	4 (1.8%)	3 (1.0%)	2 (0.7%)
Ethnicity						
Hispanic or Latino	132 (45.8%)	83 (37.6%)	129 (44.3%)	87 (39.5%)	121 (42.0%)	130 (45.6%)
Not Hispanic or Latino	130 (45.1%)	123 (55.7%)	138 (47.4%)	116 (52.7%)	137 (47.6%)	135 (47.4%)
Unknown/not reported	26 (9.0%)	15 (6.8%)	24 (8.2%)	17 (7.7%)	30 (10.4%)	20 (7.0%)
SARS-CoV-2 serostatus at baseline ^1^						
Positive	133 (46.2%)	86 (38.9%)	146 (50.2%)	84 (38.2%)	136 (47.2%)	147 (51.6%)
Negative	153 (53.1%)	135 (61.1%)	143 (49.1%)	134 (60.9%)	148 (51.4%)	135 (47.4%)
Missing	2 (0.7%)	0	2 (0.7%)	2 (0.9%)	4 (1.4%)	3 (1.1%)

Abbreviations: SD, standard deviation. ^1^ Based on central S- and N-serology testing, with a subject declared seropositive if either one was positive and negative if both were negative.

**Table 2:** Participants with solicited local adverse events (at the vaccination site) after each dose (Full analysis set—main and sub study).

	Ad26.COV2.S 9 × 10^10^	Ad26.COV2.S 7 × 10^10^	Ad26.COV2.S 5 × 10^10^	Ad26.COV2.S 3.5 × 10^10^	Ad26.COV2.S 2.5 × 10^10^	Ad26.COV2.S 1.25 × 10^10^
**Post-dose 1**	288	221	291	220	288	285
Erythema						
Any	3 (1.0%)	5 (2.3%)	7 (2.4%)	2 (0.9%)	1 (0.3%)	2 (0.7%)
Grade 1	2 (0.7%)	5 (2.3%)	4 (1.4%)	2 (0.9%)	0	2 (0.7%)
Grade 2	1 (0.3%)	0	2 (0.7%)	0	0	0
Grade 3	0	0	1 (0.3%)	0	1 (0.3%)	0
Pain						
Any	178 (61.8%)	142 (64.3%)	161 (55.3%)	137 (62.3%)	155 (53.8%)	131 (46.0%)
Grade 1	117 (40.6%)	100 (45.2%)	116 (39.9%)	113 (51.4%)	124 (43.1%)	108 (37.9%)
Grade 2	58 (20.1%)	39 (17.6%)	41 (14.1%)	21 (9.5%)	31 (10.8%)	20 (7.0%)
Grade 3	3 (1.0%)	3 (1.4%)	4 (1.4%)	3 (1.4%)	0	3 (1.1%)
Swelling						
Any	5 (1.7%)	6 (2.7%)	8 (2.7%)	2 (0.9%)	2 (0.7%)	8 (2.8%)
Grade 1	4 (1.4%)	5 (2.3%)	7 (2.4%)	2 (0.9%)	2 (0.7%)	8 (2.8%)
Grade 2	1 (0.3%)	1 (0.5%)	1 (0.3%)	0	0	0
Grade 3	0	0	0	0	0	0
**Post-dose 2**	264	200	269	199	270	258
Erythema						
Any	0	0	2 (0.7%)	2 (1.0%)	1 (0.4%)	3 (1.2%)
Grade 1	0	0	2 (0.7%)	2 (1.0%)	1 (0.4%)	3 (1.2%)
Grade 2	0	0	0	0	0	0
Grade 3	0	0	0	0	0	0
Pain						
Any	129 (48.9%)	100 (50.0%)	123 (45.7%)	94 (47.2%)	110 (40.7%)	103 (39.9%)
Grade 1	91 (34.5%)	78 (39.0%)	94 (34.9%)	76 (38.2%)	84 (31.1%)	88 (34.1%)
Grade 2	36 (13.6%)	20 (10.0%)	27 (10.0%)	18 (9.0%)	25 (9.3%)	14 (5.4%)
Grade 3	2 (0.8%)	2 (1.0%)	2 (0.7%)	0	1 (0.4%)	1 (0.4%)
Swelling						
Any	3 (1.1%)	3 (1.5%)	4 (1.5%)	1 (0.5%)	1 (0.4%)	1 (0.4%)
Grade 1	2 (0.8%)	1 (0.5%)	3 (1.1%)	1 (0.5%)	0	1 (0.4%)
Grade 2	1 (0.4%)	2 (1.0%)	1 (0.4%)	0	1 (0.4%)	0
Grade 3	0	0	0	0	0	0

Pain: Grade 1 = Aware of symptoms but easily tolerated, does not interfere with activity, discomfort only to touch; Grade 2 = Notable symptoms, requires modification in activity or use of medications, discomfort with movement; Grade 3 = Incapacitating symptoms, inability to do work, school, or usual activities, use of narcotic pain reliever. Erythema and selling: Grade 1 = 25–50 mm; Grade 2 = 51–100 mm; Grade 3 = >100 mm.

**Table 3:** Participants with solicited systemic adverse events after each dose (Full analysis set—main and sub study).

	Ad26.COV2.S 9 × 10^10^	Ad26.COV2.S 7 × 10^10^	Ad26.COV2.S 5 × 10^10^	Ad26.COV2.S 3.5 × 10^10^	Ad26.COV2.S 2.5 × 10^10^	Ad26.COV2.S 1.25 × 10^10^
**Post-dose 1**	288	221	291	220	288	285
Fatigue						
Any	152 (52.8%)	127 (57.5%)	124 (42.6%)	96 (43.6%)	102 (35.4%)	106 (37.2%)
Grade 1	70 (24.3%)	59 (26.7%)	72 (24.7%)	52 (23.6%)	53 (18.4%)	63 (22.1%)
Grade 2	70 (24.3%)	47 (21.3%)	48 (16.5%)	38 (17.3%)	47 (16.3%)	38 (13.3%)
Grade 3	12 (4.2%)	21 (9.5%)	4 (1.4%)	6 (2.7%)	2 (0.7%)	5 (1.8%)
Headache						
Any	159 (55.2%)	127 (57.5%)	121 (41.6%)	85 (38.6%)	108 (37.5%)	101 (35.4%)
Grade 1	79 (27.4%)	55 (24.9%)	67 (23.0%)	49 (22.3%)	63 (21.9%)	67 (23.5%)
Grade 2	71 (24.7%)	56 (25.3%)	54 (18.6%)	31 (14.1%)	43 (14.9%)	31 (10.9%)
Grade 3	9 (3.1%)	16 (7.2%)	0	5 (2.3%)	2 (0.7%)	3 (1.1%)
Myalgia						
Any	157 (54.5%)	110 (49.8%)	122 (41.9%)	94 (42.7%)	95 (33.0%)	91 (31.9%)
Grade 1	92 (31.9%)	54 (24.4%)	72 (24.7%)	60 (27.3%)	66 (22.9%)	60 (21.1%)
Grade 2	58 (20.1%)	42 (19.0%)	45 (15.5%)	30 (13.6%)	25 (8.7%)	27 (9.5%)
Grade 3	7 (2.4%)	14 (6.3%)	5 (1.7%)	4 (1.8%)	4 (1.4%)	4 (1.4%)
Nausea						
Any	72 (25.0%)	66 (29.9%)	53 (18.2%)	47 (21.4%)	48 (16.7%)	46 (16.1%)
Grade 1	54 (18.8%)	48 (21.7%)	36 (12.4%)	28 (12.7%)	38 (13.2%)	30 (10.5%)
Grade 2	16 (5.6%)	17 (7.7%)	17 (5.8%)	17 (7.7%)	7 (2.4%)	14 (4.9%)
Grade 3	2 (0.7%)	1 (0.5%)	0	2 (0.9%)	3 (1.0%)	2 (0.7%)
Pyrexia						
Any	56 (19.4%)	35 (15.8%)	27 (9.3%)	16 (7.3%)	17 (5.9%)	11 (3.9%)
Grade 1	27 (9.4%)	15 (6.8%)	14 (4.8%)	11 (5.0%)	9 (3.1%)	6 (2.1%)
Grade 2	21 (7.3%)	18 (8.1%)	8 (2.7%)	3 (1.4%)	7 (2.4%)	5 (1.8%)
Grade 3	8 (2.8%)	2 (0.9%)	5 (1.7%)	2 (0.9%)	1 (0.3%)	0
**Post-Dose 2**	264	200	269	199	270	258
Fatigue						
Any	96 (36.4%)	75 (37.5%)	83 (30.9%)	68 (34.2%)	71 (26.3%)	72 (27.9%)
Grade 1	56 (21.2%)	38 (19.0%)	56 (20.8%)	43 (21.6%)	43 (15.9%)	47 (18.2%)
Grade 2	34 (12.9%)	32 (16.0%)	24 (8.9%)	22 (11.1%)	26 (9.6%)	21 (8.1%)
Grade 3	6 (2.3%)	5 (2.5%)	3 (1.1%)	3 (1.5%)	2 (0.7%)	4 (1.6%)
Headache						
Any	100 (37.9%)	75 (37.5%)	74 (27.5%)	64 (32.2%)	67 (24.8%)	64 (24.8%)
Grade 1	56 (21.2%)	45 (22.5%)	49 (18.2%)	37 (18.6%)	39 (14.4%)	43 (16.7%)
Grade 2	34 (12.9%)	26 (13.0%)	23 (8.6%)	25 (12.6%)	23 (8.5%)	21 (8.1%)
Grade 3	10 (3.8%)	4 (2.0%)	2 (0.7%)	2 (1.0%)	5 (1.9%)	0
Myalgia						
Any	103 (39.0%)	65 (32.5%)	66 (24.5%)	65 (32.7%)	64 (23.7%)	60 (23.3%)
Grade 1	73 (27.7%)	39 (19.5%)	39 (14.5%)	44 (22.1%)	41 (15.2%)	36 (14.0%)
Grade 2	28 (10.6%)	24 (12.0%)	26 (9.7%)	17 (8.5%)	19 (7.0%)	21 (8.1%)
Grade 3	2 (0.8%)	2 (1.0%)	1 (0.4%)	4 (2.0%)	4 (1.5%)	3 (1.2%)
Nausea						
Any	51 (19.3%)	33 (16.5%)	26 (9.7%)	24 (12.1%)	30 (11.1%)	34 (13.2%)
Grade 1	33 (12.5%)	26 (13.0%)	18 (6.7%)	16 (8.0%)	18 (6.7%)	23 (8.9%)
Grade 2	16 (6.1%)	6 (3.0%)	6 (2.2%)	5 (2.5%)	8 (3.0%)	11 (4.3%)
Grade 3	2 (0.8%)	1 (0.5%)	2 (0.7%)	3 (1.5%)	4 (1.5%)	0
Pyrexia						
Any	21 (8.0%)	7 (3.5%)	10 (3.7%)	11 (5.5%)	8 (3.0%)	6 (2.3%)
Grade 1	14 (5.3%)	3 (1.5%)	5 (1.9%)	5 (2.5%)	5 (1.9%)	3 (1.2%)
Grade 2	5 (1.9%)	4 (2.0%)	5 (1.9%)	4 (2.0%)	0	2 (0.8%)
Grade 3	2 (0.8%)	0	0	2 (1.0%)	3 (1.1%)	1 (0.4%)

Nausea: Grade 1 = Minimal symptoms, causes minimal or no interference with work, school, or selfcare activities; Grade 2 = Notable symptoms, requires modification in activity or use of medications, does not result in loss of work, school, or cancelation of social activities; Grade 3 = Incapacitating symptoms, requires bed rest and/or results in loss of work, school, or cancelation of social activities. Fever: Grade 1 = 38.0–38.4 °C; Grade 2 = 38.5–38.9 °C; Grade 3 = 39.0–40.0 °C. Other symptoms: Grade 1 = Minimal symptoms causing no or minimal interference with usual social and functional activities; Grade 2 = Notable symptoms causing greater than minimal interference with usual social and functional activities (may require use of medications); Grade 3 = Severe symptoms causing inability to perform usual social and functional activities and requires medical intervention (may require use of narcotic pain reliever).

## Data Availability

Janssen has an agreement with the Yale Open Data Access (YODA) Project to serve as the independent review panel for the evaluation of requests for clinical study reports and participant-level data from investigators and physicians for scientific research that will advance medical knowledge and public health. Data will be made available following publication and approval by YODA of any formal requests with a defined analysis plan. For more information on this process or to make a request, please visit the YODA Project site at http://yoda.yale.edu. The data sharing policy of Janssen Pharmaceutical Companies of Johnson & Johnson is available at https://www.janssen.com/clinical-trials/transparency (accessed 29 September 2024).

## Supplementary Materials

The following supporting information can be downloaded at https://www.mdpi.com/article/10.3390/vaccines12101136/s1: Institutional Review Boards and Ethics Committees. Inclusion and exclusion criteria. Assay methods. Table S1: Spike-binding antibody levels as measured by S-ELISA (EU/mL) at all time points per regimen (per protocol immunogenicity analysis set). Table S2: Spike-binding antibody levels, as measured by S-ELISA (EU/mL) at all time points in participants seronegative for SARS-CoV-2 at baseline (non-inferiority analysis set). Table S3: Neutralizing antibody levels to the reference strain (D614G) as measured by pseudotyped virus neutralization assay up to Week 32 in a subset* of participants per dose level (per protocol immunogenicity analysis set). Table S4: Neutralizing antibody levels to the reference strain (D614G) as measured by pseudotyped virus neutralization assay up to Week 32 in a subset* of participants seronegative at baseline, per dose level (non-inferiority analysis set). Table S5: Unsolicited adverse events reported by at least 2% of participants in any group within 28 days after any vaccination (full analysis set). Table S6: Serious adverse events reported over the entire study duration (full analysis set). Figure S1: Neutralizing antibody levels to the reference strain (D614G) as measured by pseudotyped virus neutralization assay up to week 32 in a subset * of participants, per dose level (A) per protocol immunogenicity analysis set, (B) non-inferiority analysis set. Figure S2: Correlation between spike-binding and neutralizing antibody levels at Day 29, 71, and Week 32.
