# Supplementary material for: Safety and Humoral Immunogenicity of Different Dose Levels of Ad26.COV2.S as a 2-Dose Regimen in COVID-19 Vaccine-Naïve Healthy Adults: A Phase 3 Randomized Clinical Trial
Source: Vaccines (Basel). 2024 Oct 3;12(10):1136. doi: 10.3390/vaccines12101136 (PMC11511116; doi:10.3390/vaccines12101136)
Supplement: Supplementary file 1 [file vaccines-12-01136-s001.zip › vaccines-3204722-supplementary.pdf]

Safety and humoral immunogenicity of different dose levels of Ad26.COV2.S as a two dose regimen in COVID-19 vaccine naïve healthy adults: a phase 3 randomized clinical trial

SUPPLEMENTARY INFORMATION

Institutional Review Boards and Ethics Committees

Country	IRB/EC	Approval Date	Approval/ Reference Number
Brazil	Santa Casa de Misericordia de Belo Horizonte (IRB00011555)	29 July 2021	4.874.227
	Universidade Federal de Minas Gerais (IRB00002198)	12 December 2021	5.174.495
	Universidade Federal do Ceara (IRB00004330)	03 November 2021	5.076.988
	Sociedade Literária e Caritativa Santo Agostinho—Hospital São José (IRB00012820)	02 September 2021	4.950.578
	Instituto de Saúde e Bem Estar da Mulher (IRB00012701)	25 August 2021	4.929.177
	Universidade Federal de Roraima/CECOR Centro Oncologico de Roraima	07 September 2021	4.956.636
	Pontificia Universidade Catolica de Campinas (IRB00006170)	15 September 2021	4.976.143
	Hospital Universitário Onofre Lopes (IRB00013721)	20 September 2021	5.009.613
	Hospital das Clínicas da Faculdade de Medicina de Ribeirão Preto (IRB00002186)	27 August 2021	4.936.476
	Universidade Iguacu—Nova Iguacu Prefeitura (IRB00005749)	21 September 2021	4.988.381
USA	Sterling IRB	19 January 2021	8625
South Africa	Pharma-Ethics	29 September 2021	210824178

Inclusion criteria

1. Participant must sign an ICF indicating that he or she understands the purpose, procedures and potential risks and benefits of the study, and is willing to participate in the study.

2. Participant is willing and able to adhere to the prohibitions and restrictions specified in this protocol.

3. Participant is 18 to 55 years of age, inclusive, on the day of signing the ICF.

4. Participant must have a BMI <35.0 kg/m2.

5. Participant must be healthy, in the investigator's clinical judgment, as confirmed by medical history, physical examination, and vital signs performed at screening. Participant may have underlying illnesses, as long as the symptoms and signs are medically controlled and not considered to be comorbidities related to an increased risk of severe COVID-19, except for smoking, which is allowed (see also exclusion criterion 21). If on medication for a condition, the medication dose must have been stable for at least 12 weeks preceding vaccination and expected to remain stable for the duration of the study. Participant will be included on the basis of physical examination, medical history, and vital signs.

6. Contraceptive (birth control) use should be consistent with local regulations regarding the acceptable methods of contraception for those participating in clinical studies. Before randomization, participants must be either:

a. Not of childbearing potential

b. Of childbearing potential and practicing a highly effective method of contraception and agrees to remain on such a method of contraception from signing the consent until 3 months after the last dose of study vaccine. Use of hormonal contraception should start at least 28 days before the 1st administration of study vaccine. The investigator should evaluate the potential for contraceptive method failure (eg, noncompliance, recently initiated) in relationship to the 1st vaccination. Highly effective methods for this study include:

1. hormonal contraception:

i. combined (estrogen and progestogen containing) hormonal contraception associated with inhibition of ovulation (oral, intravaginal, or transdermal)

ii. progestogen-only hormonal contraception associated with inhibition of ovulation (oral, injectable, or implantable)

2. intrauterine device;

3. intrauterine hormone-releasing system;

4. bilateral tubal occlusion/ligation procedure;

5. vasectomized partner (the vasectomized partner should be the sole partner for that participant);

6. sexual abstinence

7. All female participants of childbearing potential must:

a. Have a negative highly sensitive urine pregnancy test at screening

b. Have a negative highly sensitive urine pregnancy test immediately on the day of and prior to each study vaccine administration.

8. Participant agrees to not donate bone marrow, blood, and blood products from the first study vaccine administration until 3 months after receiving the last dose of study vaccine.

9. Participant must be willing to provide verifiable identification, has means to be contacted and to contact the investigator during the study.

Exclusion Criteria

1. Participant has a clinically significant acute illness (this does not include minor illnesses such as diarrhea or mild upper respiratory tract infection) or temperature ≥38.0ºC (100.4°F) within 24 hours prior to the planned first dose of study vaccine; randomization at a later date is permitted at the discretion of the investigator and after consultation with the sponsor.

2. Participant has a history of malignancy within 5 years before screening (exceptions are squamous and basal cell carcinomas of the skin and carcinoma in situ of the cervix, or malignancy, which is considered cured with minimal risk of recurrence).

3. Participant has a known or suspected allergy or history of anaphylaxis or other serious adverse reactions to vaccines or their excipients (including specifically the excipients of the study vaccine).

4. Participant has abnormal function of the immune system resulting from:

a. Clinical conditions (eg, autoimmune disease, potential immune mediated disease or known or suspected immunodeficiency, chronic kidney disease [with dialysis]) expected to have an impact on the immune response of the study vaccine. Participants with clinical conditions stable under non-immunomodulator treatment eg, autoimmune thyroiditis, autoimmune inflammatory rheumatic disease such as rheumatoid arthritis) may be enrolled at the discretion of the investigator. Non-immunomodulator treatment is allowed as well as steroids at a non-immunosuppressive dose or route of administration.

b. Chronic or recurrent use of systemic corticosteroids within 6 months before administration of study vaccine and during the study. A substantial immunosuppressive steroid dose is considered to be =>2 weeks of daily receipt of 20 mg prednisone or equivalent

Note: Ocular, topical or inhaled steroids are allowed.

c. Administration of antineoplastic and immunomodulating agents or radiotherapy within 6 months before administration of study vaccine and during the study.

5. Participant has a history of any neurological disorders or seizures including Guillain-Barré syndrome, with the exception of febrile seizures during childhood.

6. Participant has a history of chronic urticaria (recurrent hives), eczema or adult atopic dermatitis.

7. Participant received treatment with immunoglobulins in the 3 months or exogenous blood products (autologous blood transfusions are not exclusionary) in the 4 months before the planned administration of the first dose of study vaccine or has any plans to receive such treatment during the study.

8. Participant received or plans to receive:

a. Licensed live attenuated vaccines –within 28 days before or after planned administration of the first or subsequent study vaccinations

b. Other licensed (not live) vaccines –within 14 days before or after planned administration of the first or subsequent study vaccinations.

9. Participant received an investigational drug (including investigational drugs for prophylaxis of COVID-19) or used an invasive investigational medical device within 30 days or received investigational Ig or monoclonal antibodies within 3 months, or received convalescent serum for COVID-19 treatment within 4 months or received an investigational vaccine within 6 months before the planned administration of the first dose of study vaccine or is currently enrolled or plans to participate in another investigational study during the course of this study.

Efforts will be made to ensure inclusion of participants who have not been previously in coronavirus studies and to prevent participants from subsequently enrolling in other coronavirus studies during their participation in this study.

The use of any coronavirus vaccine (licensed or investigational) other than Ad26.COV2.S is disallowed at any time prior to vaccination (see also Exclusion Criterion 19) and during the study except under the conditions described elsewhere in the protocol.

10. Participant is a woman who is pregnant or planning to become pregnant within 3 months after the last dose of study vaccine.

11. Participant has a history of an underlying clinically significant acute or chronic medical condition or physical examination findings for which, in the opinion of the investigator, participation would not be in the best interest of the participant (eg, compromise the wellbeing) or that could prevent, limit, or confound the protocol-specified assessments.

12. Participant had surgery requiring hospitalization (defined as inpatient stay for longer than 24 hours or overnight stay), within 12 weeks before vaccination, or will not have fully recovered from surgery requiring hospitalization or has surgery requiring hospitalization planned during the time the participant is expected to participate in the study or within 6 months after the last dose of study vaccine administration.

13. Participant has a contraindication to IM injections and blood draws eg, bleeding disorders.

14. Participant is an employee of the investigator or study site, with direct involvement in the proposed study or other studies under the direction of that investigator or study site, as well as family members of the employees or the investigator, or an employee of the sponsor.

15. Participant has chronic active hepatitis B or hepatitis C infection per medical history.

16. Participant has had major psychiatric illness or drug or alcohol abuse which in the investigator’s opinion would compromise the participant’s safety or compliance with the

study procedures.

17. Participant cannot communicate reliably with the investigator.

18. Participant who, in the opinion of the investigator, is unlikely to adhere to the requirements of the study or is unlikely to complete the full course of vaccination and observation.

19. Participant previously received a coronavirus vaccine.

20. Participant has a positive diagnostic test result for past (serological testing) or current (PCR based viral RNA detection) SARS-CoV-2 infection at screening in the main study.

Note: For participants in the sub study, a positive diagnostic test for past SARS-CoV-2 testing (serological testing) does not exclude the participant from the study as a target of approximately 40 seronegative and 20 seropositive participants per group (Group 1, 3, 5 and 6) will be enrolled in the sub study. Seropositive participants are not excluded up to the point when 20 seropositive participants are recruited only.

21. Participants with comorbidities that are or might be associated with an increased risk of progression to severe COVID-19, ie, participants with moderate-to-severe asthma; chronic lung diseases such as COPD (including emphysema and chronic bronchitis), idiopathic pulmonary fibrosis and cystic fibrosis; diabetes (including type 1 or type 2); serious heart conditions, including heart failure, coronary artery disease, congenital heart disease, cardiomyopathies, and (pulmonary) hypertension or high blood pressure; obesity (BMI ≥ 35 kg/m2); chronic liver disease, including cirrhosis; sickle cell disease; thalassemia; cerebrovascular disease; neurologic conditions (dementia); end stage renal disease; organ transplantation; cancer; and other immunodeficiencies; hepatitis B infection; and sleep apnea, and participants who live in nursing homes or long-term care facilities. This list is consistent with the list of conditions that increase the risk of progression to severe COVID-19 available at the CDC website at the time of writing of the, except for smoking, which is allowed. Participants may have hypertension of mild severity, as long as it is stable and medically controlled as defined by no change in medication over the past 6 months (except for issues of tolerability or use of similar drug with same mechanism of action, eg, thiazides, Beta blockers, Alpha blockers at the same effective dose).

22. Participant who is currently working in an occupation with a high risk of exposure to SARSCoV-2 infection (eg, health care worker or emergency response personnel who work in close contact with SARSCoV-2 infected patients) or considered at the investigator’s discretion to be at increased risk to acquire COVID-19 for any other reason.

23. Participant who has had a known exposure to an individual with confirmed COVID-19 or SARS-CoV-2 infection within 2 weeks of screening.

24. History of confirmed SARS or MERS.

25. History of capillary leak syndrome

26. History of TTS or heparin-induced thrombocytopenia and thrombosis (HITT)

Assay methods

Spike Protein Enzyme-linked Immunosorbent Assay (S-ELISA). SARS-CoV-2 prefusion conformation S protein specific binding antibody concentrations were determined using the human SARS-CoV-2 Pre-Spike IgG ELISA, an indirect ELISA which is based on the antibody/antigen interactions. The SARS-CoV-2 antigen used is a stabilized prefusion S protein ((2P), Δfurin, T4 foldon, His-Tag), derived from the first clinical isolate of the Wuhan strain (Wuhan, 2019, whole genome sequence NC_045512), produced in ES-293 cells. The ELISA was developed, qualified, and validated (as of 30 November 2020) for human serum at Nexelis, Laval, Canada. In brief, purified SARS-CoV-2 Pre-Spike Antigen was adsorbed to the wells of a microplate and diluted serum samples (test samples, standard, and quality controls) were added. Unbound sample was washed away, and enzyme-conjugated anti-human IgG added. After washing excess conjugate away, 3,3′,5,5′-tetramethylbenzidine colorimetric substrate was added. After the established time period, the reaction was stopped. A reference standard on each test plate was used to quantify the amount of antibodies against SARS-CoV-2 Pre-Spike in the sample according to the unit assigned by the standard (ELISA Laboratory Unit per milliliter: EU/mL). The LLOQ and ULOQ of the assay were 50.3 and 58,158.1 EU/mL, respectively.

SARS-CoV-2 Neutralization (psVNA). Pseudotyped virus neutralization assays were validated and performed by Monogram Biosciences, Labcorp. The measurement of neutralizing antibody activity was performed by generating HIV-1 pseudovirions that expressed the SARS-CoV-2 S protein from the reference strain (with D614G mutation). The pseudoviruses were prepared by co-transfecting HEK293 producer cells with an HIV-1 genomic vector and a SARS-CoV-2 envelope expression vector. Neutralizing antibody activity was measured by assessing the inhibition of luciferase activity in HEK293 target cells expressing the ACE2 receptor following pre-incubation of the pseudovirions with serially diluted serum samples. The expression of luciferase activity in target cells was inhibited by the presence of functional anti-SARS-CoV-2 antibodies with neutralizing activity. Data were displayed by plotting the percent inhibition of luciferase activity versus log10 reciprocal of the serum dilution and antibody titers were reported as the reciprocal of the serum dilution/concentration conferring 50% inhibition (IC50) of pseudovirus infection. To ensure that the neutralizing activity measured was specific for SARS-CoV-2, each test sample was also assessed using a non-specific pseudovirus (specificity control) that expressed a non-reactive envelope protein of one or more unrelated viruses (eg, avian influenza virus). The LLOQ and ULOQ of the assay were an IC50 of 75 and 12,936, respectively.

Supplementary results

Table S1 Spike-binding antibody levels as measured by S-ELISA (EU/mL) at all time points per regimen (per protocol immunogenicity analysis set)

		Ad26.COV2.S dose level (vp)					
		**9×10^{10}**	**7×10^{10}**	**5×10^{10}**	**3.5×10^{10}**	**2.5×10^{10}**	**1.25×10^{10}**
Baseline	N	275	212	281	212	277	270
	GMC (95% CI)	147 (111;194)	93 (71;120)	159 (121;209)	93 (71;122)	160 (120;212)	193 (144;259)
Day 29	N	249	191	250	189	243	251
	GMC (95% CI)	2351 (1849;2989)	1714 (1305;2250)	2189 (1726;2775)	1377 (1062;1786)	1626 (1255;2106)	1976 (1521;2566)
	GMI (95% CI) from Baseline	11.3 (9.3;13.7)	12.7 (10.5;15.4)	9.9 (8.2;12.1)	10.1 (8.3;12.3)	7.6 (6.3;9.3)	7.3 (6;8.8)
Day 57	N	195	151	198	149	195	199
	GMC (95% CI)	2431 (1906;3100)	1623 (1225;2152)	2001 (1569;2551)	1463 (1113;1925)	1674 (1283;2185)	2157 (1647;2825)
	GMI (95% CI) from Baseline	10.3 (8.3;12.8)	12.9 (10.5;15.9)	8.5 (6.9;10.5)	12.5 (10.3;15.3)	7.3 (5.9;9.1)	6.7 (5.4;8.2)
Day 71	N	171	130	180	134	172	184
	GMC (95% CI)	4997 (4145;6023)	3757 (3030;4659)	3855 (3185;4666)	3460 (2781;4304)	3641 (2969;4465)	3905 (3113;4897)
	GMI (95% CI) from Baseline	21.9 (17;28.2)	30.3 (24.7;37.1)	17.9 (14;22.8)	27.8 (21.9;35.3)	16.3 (12.5;21.3)	13 (10;16.8)
	GMI (95% CI) from Day 57	2.2 (1.9;2.5)	2.5 (2.1;2.9)	2 (1.8;2.3)	2.4 (2;2.7)	2.2 (1.8;2.6)	2 (1.7;2.3)
Week 32	N	97	71	110	80	106	119
	GMC (95% CI)	3514 (2617;4717)	2727 (1933;3845)	2565 (1935;3400)	1886 (1331;2671)	3231 (2385;4378)	3038 (2201;4193)
	GMI (95% CI) from Baseline	7.5 (5.2;10.9)	11.5 (8.1;16.3)	6 (4.4;8.1)	12.1 (8.8;16.7)	6.7 (4.7;9.5)	4.9 (3.6;6.7)
	GMI (95% CI) from Day 57	0.9 (0.7;1)	0.8 (0.6;0.9)	0.7 (0.6;0.8)	1 (0.8;1.2)	0.9 (0.7;1.1)	0.8 (0.7;1)
Week 60	N	45	33	42	37	45	52
	GMC (95% CI)	4968 (3119;7915)	5010 (3146;7981)	2363 (1448;3857)	2191 (1224;3921)	2769 (1776;4317)	3664 (2169;6190)
	GMI (95% CI) from Baseline	7.9 (4.7;13.4)	16.5 (9.3;29.3)	4.4 (2.6;7.3)	8.5 (5.3;13.8)	3.9 (2.3;6.4)	4 (2.5;6.2)
	GMI (95% CI) from Day 57	0.7 (0.5;1.1)	1 (0.6;1.6)	0.7 (0.5;0.9)	0.6 (0.4;0.8)	0.7 (0.5;0.8)	0.7 (0.5;0.9)

EU/mL. ELISA Units per mL; CI, confidence interval; GMC, Geometric mean concentrations; GMI, geometric mean fold increase; N, number of subjects with data; vp, viral particles

Table S2 Spike-binding antibody levels, as measured by S-ELISA (EU/mL) at all time points in participants seronegative for SARS-CoV-2 at baseline (non-inferiority analysis set)

		**Ad26.COV2.S dose level (vp)**					
		**9×10^{10}**	**7×10^{10}**	**5×10^{10}**	**3.5×10^{10}**	**2.5×10^{10}**	**1.25×10^{10}**
Baseline	N	146	127	139	131	144	129
	GMC (95% CI)	<LLOQ	<LLOQ	<LLOQ	<LLOQ	<LLOQ	<LLOQ
Day 29	N	133	116	128	120	130	120
	GMC (95% CI)	530 (440;639)	502 (414;609)	479 (398;576)	456 (380;548)	326 (273;390)	300 (243;370)
	GMI (95% CI) from Baseline	10.7 (8.9;12.8)	10 (8.3;12.1)	9.6 (8;11.5)	9.1 (7.6;10.9)	6.6 (5.6;7.9)	6.2 (5.1;7.5)
Day 57	N	104	98	101	101	105	92
	GMC (95% CI)	649 (540;780)	580 (468;720)	550 (451;670)	583 (474;717)	404 (334;488)	380 (303;477)
	GMI (95% CI) from Baseline	12.9 (10.7;15.5)	11.7 (9.5;14.4)	10.9 (9;13.3)	11.8 (9.6;14.4)	8.1 (6.8;9.8)	7.6 (6;9.5)
Day 71	N	96	87	94	90	94	90
	GMC (95% CI)	2292 (1932;2719)	1979 (1640;2388)	1818 (1480;2234)	2018 (1617;2519)	1666 (1353;2051)	1403 (1101;1788)
	GMI (95% CI) from Baseline	45.6 (38.4;54)	39.3 (32.6;47.5)	36.1 (29.4;44.4)	40.1 (32.1;50.1)	33.1 (26.9;40.8)	27.9 (21.9;35.5)
	GMI (95% CI) from Day 57	3.5 (3;4.1)	3.5 (3;4.2)	3.3 (2.8;3.9)	3.4 (2.8;4.1)	4 (3.2;5)	4 (3.2;5)
Week 32	N	34	30	31	43	34	32
	GMC (95% CI)	855 (577;1266)	860 (547;1353)	600 (412;873)	693 (473;1015)	799 (469;1362)	383 (220;666)
	GMI (95% CI) from Baseline	17 (11.5;25.2)	17.1 (10.9;26.9)	11.9 (8.2;17.4)	14.2 (10;20.3)	16.2 (9.7;27.2)	8.1 (4.8;13.6)
	GMI (95% CI) from Day 57	1.4 (0.9;2.1)	1.1 (0.7;1.7)	0.8 (0.7;1)	1.3 (1;1.7)	1.7 (0.9;3.2)	1.3 (0.8;2.4)
Week 60	N	14	10	11	15	15	12
	GMC (95% CI)	1015 (474;2174)	1847 (539;6332)	852 (353;2053)	423 (189;949)	527 (317;877)	236 (83;672)
	GMI (95% CI) from Baseline	20.2 (9.4;43.2)	36.7 (10.7;125.9)	16.9 (7;40.8)	8.4 (3.8;18.9)	10.5 (6.3;17.4)	5.3 (2;13.6)
	GMI (95% CI) from Day 57	1.1 (0.5;2.3)	3.7 (1;12.8)	0.8 (0.3;1.9)	0.7 (0.4;1.4)	1 (0.6;1.6)	0.7 (0.2;2.3)

EU/mL. ELISA Units per mL; CI, confidence interval; GMC, Geometric mean concentrations; GMI, geometric mean fold increase; LLOQ, lower limit of quantitation; N, number of subjects with data; vp, viral particles

Table S3 Neutralizing antibody levels to the reference strain (D614G) as measured by pseudotyped virus neutralization assay up to Week 32 in a subset* of participants per dose level (per protocol immunogenicity analysis set)

		Ad26.COV2.S dose level (vp)					
		**9×10^10**	**7×10^10**	**5×10^10**	**3.5×10^10**	**2.5×10^10**	**1.25×10^10**
Baseline	N	164	98	166	99	166	164
	GMT (95% CI)	105 (78;141)	<LLOQ	104 (79;136)	<LLOQ	118 (87;159)	132 (96;180)
Day 29	N	160	93	154	98	157	155
	GMT (95% CI)	419 (301;584)	125 (93;169)	352 (252;491)	113 (86;147)	307 (220;429)	367 (259;521)
	GMI (95% CI) from Baseline	3 (2.4;3.7)	2 (1.7;2.5)	2.7 (2.2;3.4)	1.9 (1.6;2.2)	2.2 (1.8;2.7)	2.2 (1.9;2.7)
Day 57	N	148	91	153	98	155	147
	GMT (95% CI)	357 (254;502)	110 (83;145)	328 (240;448)	130 (100;168)	273 (198;378)	324 (227;462)
	GMI (95% CI) from Baseline	2.5 (2;3.1)	1.9 (1.5;2.3)	2.5 (2;3)	2 (1.7;2.4)	2.1 (1.7;2.6)	2 (1.7;2.4)
Day 71	N	138	84	139	91	142	139
	GMT (95% CI)	1038 (809;1332)	481 (382;604)	750 (577;975)	492 (366;659)	737 (565;962)	722 (539;965)
	GMI (95% CI) from Baseline	6.1 (4.9;7.6)	6 (4.9;7.3)	5.3 (4.2;6.6)	6.3 (4.9;8.1)	4.8 (3.8;6)	3.7 (3;4.7)
	GMI (95% CI) from Day 57	2.6 (2.2;3.1)	3.2 (2.7;3.9)	2.1 (1.8;2.5)	3.1 (2.5;3.9)	2.3 (1.9;2.8)	1.9 (1.6;2.3)
Week 32	N	73	33	78	42	82	83
	GMT (95% CI)	848 (554;1297)	254 (149;431)	599 (405;888)	227 (150;343)	819 (540;1242)	858 (545;1350)
	GMI (95% CI) from Baseline	3.2 (2.2;4.7)	3.4 (2.2;5.2)	3 (2.2;4)	3.6 (2.5;5)	3.2 (2.2;4.6)	2.8 (2.1;3.8)
	GMI (95% CI) from Day 57	1.3 (0.9;1.8)	1.4 (0.9;2.3)	1 (0.8;1.3)	2 (1.3;2.8)	1.3 (0.9;1.7)	1.3 (1;1.7)

CI, confidence interval; GMT, Geometric mean titer; GMI, geometric mean fold increase; LLOQ, lower limit of quantitation; N, number of subjects with data; vp, viral particles

Neutralizing titers are expressed as IC50 - 50% inhibitory concentration.

*100 participants per group in the main study and all participants in the sub-study.

Table S4 Neutralizing antibody levels to the reference strain (D614G) as measured by pseudotyped virus neutralization assay up to Week 32 in a subset* of participants seronegative at baseline, per dose level (non-inferiority analysis set)

		**Ad26.COV2.S dose level (vp)**					
		**9×10^{10}**	**7×10^{10}**	**5×10^{10}**	**3.5×10^{10}**	**2.5×10^{10}**	**1.25×10^{10}**
Baseline	N	94	89	90	91	96	87
	GMT (95% CI)	<LLOQ	<LLOQ	<LLOQ	<LLOQ	<LLOQ	<LLOQ
Day 29	N	93	86	87	90	93	84
	GMT (95% CI)	109 (84;143)	106 (81;139)	88 (<LLOQ;114)	93 (<LLOQ;116)	86 (<LLOQ;109)	<LLOQ (<LLOQ;91)
	GMI (95% CI) from Baseline	2 (1.7;2.5)	2 (1.6;2.5)	1.8 (1.4;2.2)	1.8 (1.5;2.1)	1.7 (1.4;2.1)	1.5 (1.3;1.8)
Day 57	N	88	86	89	90	93	82
	GMT (95% CI)	98 (77;126)	95 (<LLOQ;124)	100 (79;127)	107 (87;131)	77 (<LLOQ;95)	<LLOQ (<LLOQ;88)
	GMI (95% CI) from Baseline	1.9 (1.5;2.2)	1.8 (1.5;2.2)	1.9 (1.6;2.3)	1.9 (1.6;2.2)	1.6 (1.3;1.8)	1.5 (1.3;1.7)
Day 71	N	84	79	85	83	87	79
	GMT (95% CI)	511 (406;644)	444 (356;555)	373 (288;483)	449 (336;600)	370 (279;491)	312 (239;407)
	GMI (95% CI) from Baseline	7.1 (5.8;8.8)	6.1 (5;7.5)	5.4 (4.3;6.8)	6.5 (5;8.4)	5.4 (4.2;7)	4.5 (3.6;5.7)
	GMI (95% CI) from Day 57	3.9 (3.2;4.7)	3.4 (2.8;4.2)	2.8 (2.3;3.4)	3.5 (2.8;4.4)	3.5 (2.7;4.4)	3 (2.4;3.8)
Week 32	N	31	29	30	39	32	30
	GMT (95% CI)	247 (149;411)	234 (134;408)	169 (111;259)	208 (137;316)	221 (122;402)	148 (82;266)
	GMI (95% CI) from Baseline	3.9 (2.5;5.9)	3.7 (2.3;6)	2.7 (1.9;3.8)	3.3 (2.3;4.7)	3.6 (2.1;6.1)	2.7 (1.6;4.4)
	GMI (95% CI) from Day 57	1.9 (1.2;3.1)	1.5 (0.9;2.6)	1.1 (0.8;1.5)	1.9 (1.3;2.7)	2 (1.1;3.5)	1.6 (0.9;2.8)

CI, confidence interval; GMT, Geometric mean titer; GMI, geometric mean fold increase; LLOQ, lower limit of quantitation; N, number of subjects with data; vp, viral particles

Neutralizing titers are expressed as IC50 - 50% inhibitory concentration.

*100 participants per group in the main study and all participants in the sub-study.

Table S5 Unsolicited adverse events reported by at least 2% of participants in any group within 28 days after any vaccination (full analysis set)

	**Ad26.COV2.S dose level (vp)**						
	**9×10^{10}**	**7×10^{10}**	**5×10^{10}**	**3.5×10^{10}**	**2.5×10^{10}**	**1.25×10^{10}**	**All participants**
At least 1 AE	65 (22.6%)	49 (22.2%)	57 (19.6%)	52 (23.6%)	58 (20.1%)	57 (20.0%)	338 (21.2%)
Headache	24 (8.3%)	11 (5.0%)	9 (3.1%)	18 (8.2%)	19 (6.6%)	20 (7.0%)	101 (6.3%)
Influenza	15 (5.2%)	15 (6.8%)	22 (7.6%)	15 (6.8%)	18 (6.3%)	15 (5.3%)	100 (6.3%)
Fatigue	15 (5.2%)	10 (4.5%)	5 (1.7%)	9 (4.1%)	5 (1.7%)	15 (5.3%)	59 (3.7%)
Myalgia	10 (3.5%)	10 (4.5%)	4 (1.4%)	12 (5.5%)	6 (2.1%)	16 (5.6%)	58 (3.6%)
Vaccination site pain	7 (2.4%)	8 (3.6%)	4 (1.4%)	3 (1.4%)	6 (2.1%)	7 (2.5%)	35 (2.2%)
Nasopharyngitis	3 (1.0%)	5 (2.3%)	5 (1.7%)	3 (1.4%)	3 (1.0%)	9 (3.2%)	28 (1.8%)
Pyrexia	7 (2.4%)	7 (3.2%)	1 (0.3%)	4 (1.8%)	4 (1.4%)	5 (1.8%)	28 (1.8%)
Nausea	6 (2.1%)	3 (1.4%)	2 (0.7%)	5 (2.3%)	5 (1.7%)	5 (1.8%)	26 (1.6%)
COVID-19	2 (0.7%)	4 (1.8%)	6 (2.1%)	5 (2.3%)	4 (1.4%)	4 (1.4%)	25 (1.6%)
Chills	11 (3.8%)	4 (1.8%)	2 (0.7%)	4 (1.8%)	4 (1.4%)	0	25 (1.6%)
Rhinitis	7 (2.4%)	3 (1.4%)	0	3 (1.4%)	6 (2.1%)	2 (0.7%)	21 (1.3%)
Vomiting	4 (1.4%)	1 (0.5%)	6 (2.1%)	1 (0.5%)	0	2 (0.7%)	14 (0.9%)
Anaemia	3 (1.0%)	1 (0.5%)	6 (2.1%)	0	3 (1.0%)	0	13 (0.8%)

Table S6 Serious adverse events reported over the entire study duration (full analysis set)

	Ad26.COV2.S dose level (vp)						
	**9×10^{10}**	**7×10^{10}**	**5×10^{10}**	**3.5×10^{10}**	**2.5×10^{10}**	**1.25×10^{10}**	**All participants**
At least 1 SAE, n (%)	13 (4.5%)	3 (1.4%)	4 (1.4%)	4 (1.8%)	3 (1.0%)	5 (1.8%)	32 (2.0%)
Appendicitis	1 (0.3%)	0	0	0	0	0	1 (0.1%)
Cellulitis	1 (0.3%)	0	0	0	0	0	1 (0.1%)
Pelvic abscess	1 (0.3%)	0	0	0	0	0	1 (0.1%)
Pyelonephritis	0	0	0	1 (0.5%)	0	0	1 (0.1%)
Pyelonephritis acute	0	0	0	1 (0.5%)	0	0	1 (0.1%)
Anxiety	1 (0.3%)	0	0	0	0	0	1 (0.1%)
Anxiety disorder	0	0	0	0	0	1 (0.4%)	1 (0.1%)
Depression	0	1 (0.5%)	0	0	0	0	1 (0.1%)
Psychotic disorder	1 (0.3%)	0	0	0	0	0	1 (0.1%)
Suicide attempt	0	0	0	0	1 (0.3%)	0	1 (0.1%)
Carotid artery dissection	0	0	0	1 (0.5%)	0	0	1 (0.1%)
Myasthenia gravis	1 (0.3%)	0	0	0	0	0	1 (0.1%)
Paraesthesia	0	0	0	0	1 (0.3%)	0	1 (0.1%)
Syncope	0	0	1 (0.3%)	0	0	0	1 (0.1%)
Ankle fracture	0	1 (0.5%)	0	0	0	0	1 (0.1%)
Gun shot wound	1 (0.3%)	0	0	0	0	0	1 (0.1%)
Stab wound	1 (0.3%)	0	0	0	0	0	1 (0.1%)
Abortion spontaneous	1 (0.3%)	0	1 (0.3%)	0	0	0	2 (0.1%)
Gestational diabetes	0	0	0	0	0	1 (0.4%)	1 (0.1%)
Hydrosalpinx	1 (0.3%)	0	0	0	0	0	1 (0.1%)
Ovarian cyst torsion	0	0	0	0	0	1 (0.4%)	1 (0.1%)
Ovarian disorder	0	0	0	0	0	1 (0.4%)	1 (0.1%)
Vaginal haemorrhage	0	1 (0.5%)	0	0	0	0	1 (0.1%)
Abdominal pain	0	0	1 (0.3%)	0	0	0	1 (0.1%)
Anal fistula	0	0	0	0	0	1 (0.4%)	1 (0.1%)
Death	0	0	0	0	0	1 (0.4%)	1 (0.1%)
Drowning	1 (0.3%)	0	0	0	0	0	1 (0.1%)
Anaemia	0	0	1 (0.3%)	0	0	0	1 (0.1%)
Myopericarditis	1 (0.3%)	0	0	0	0	0	1 (0.1%)
Cholelithiasis	0	0	1 (0.3%)	0	0	0	1 (0.1%)
Intervertebral disc protrusion	0	0	0	1 (0.5%)	0	0	1 (0.1%)
Tongue neoplasm malignant stage unspecified	1 (0.3%)	0	0	0	0	0	1 (0.1%)
Nephrolithiasis	0	0	0	1 (0.5%)	0	0	1 (0.1%)
Pleural effusion	0	0	0	0	1 (0.3%)	0	1 (0.1%)

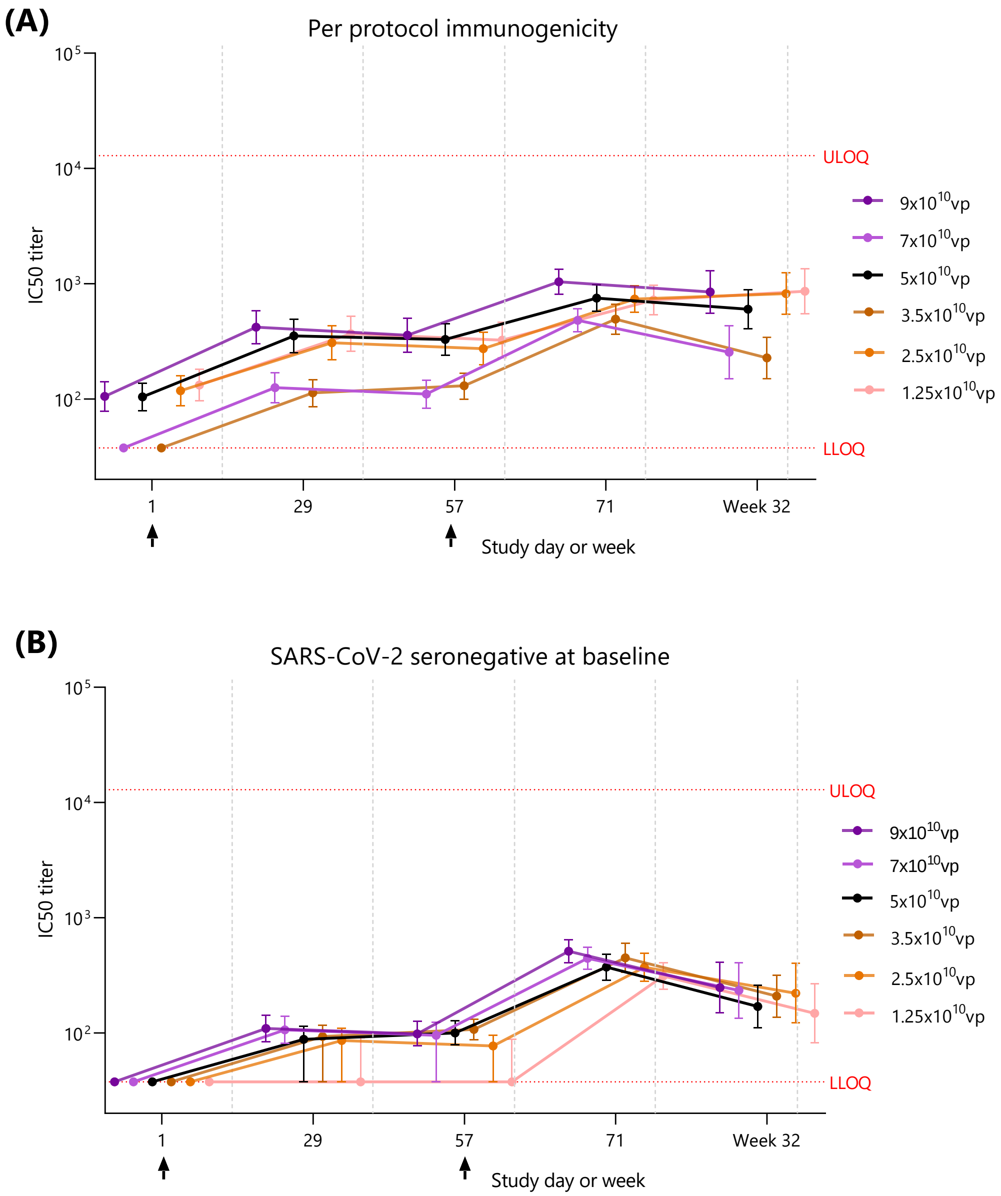

Figure S1 Neutralizing antibody levels to the reference strain (D614G) as measured by pseudotyped virus neutralization assay up to week 32 in a subset* of participants, per dose level A) per protocol immunogenicity analysis set, B) non-inferiority analysis set

LLOQ, lower limit of quantitation; N, number of subjects with data; psVNA, pseudotyped virus neutralization assay; ULOQ, upper limit of quantitation; vp, viral particles
IC50 - 50% inhibitory concentration.
Geometric mean titers and 95% confidence intervals are shown as dots and error bars, respectively. The X-axis is not continuous. Black arrows indicate Ad26.COV2.S vaccination, and red lines indicate the assay LLOQ and ULOQ (75 IC50 titer and 12936 IC50 titer, respectively).
*100 participants per group in the main study and all participants in the sub-study.

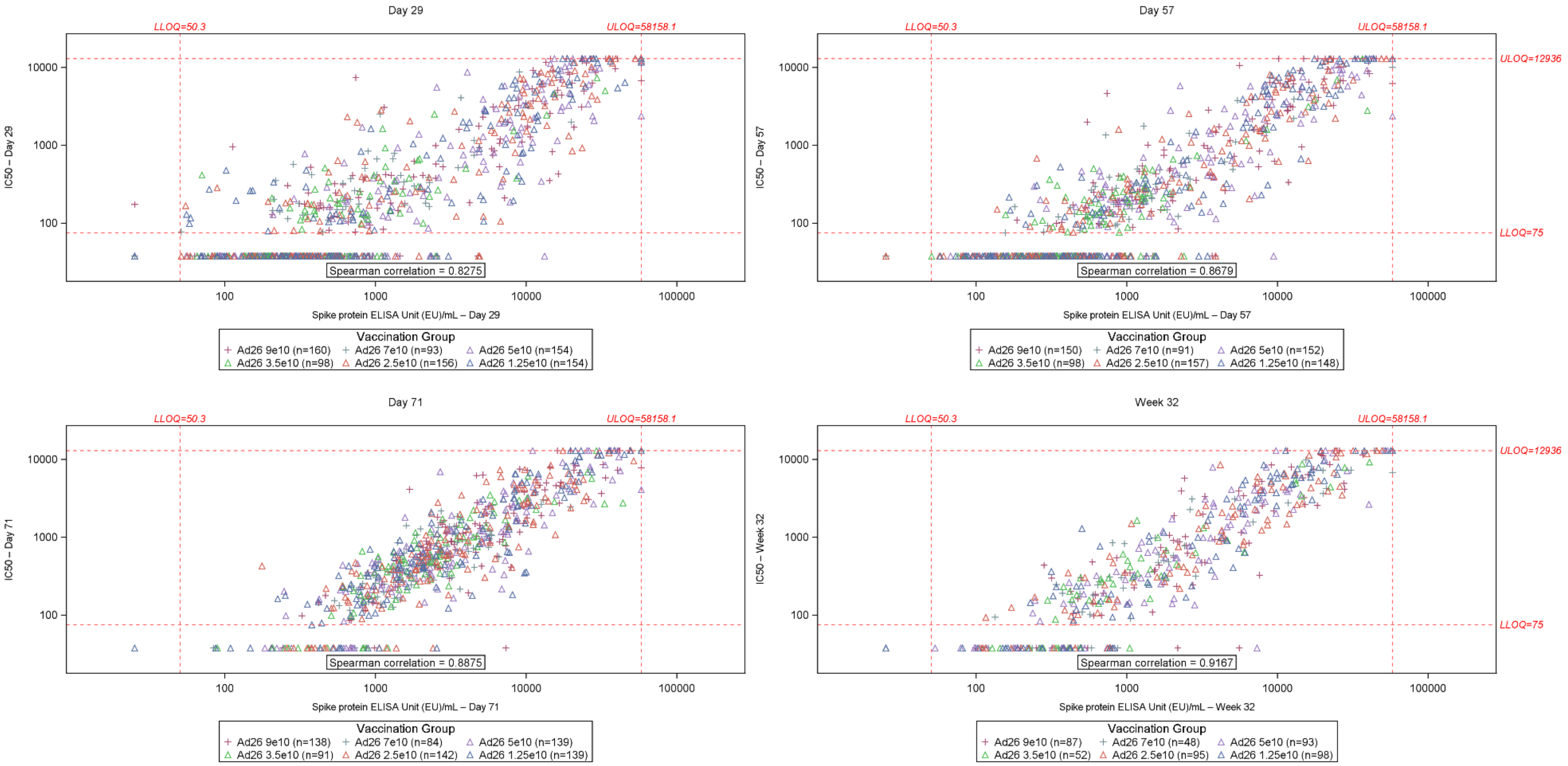

Figure S2 Correlation between Spike-binding and neutralizing antibody levels at Day 29, 57, 71, and Week 32

IC50, 50% inhibitory concentration; LOD, limit of detection; LLOQ, lower limit of quantification; ULOQ, upper limit of quantification, Spearman correlation coefficients are indicated at each time point. LLOQ and ULOQ for each assay are indicated with red lines.
